# Responsibility is not required for authorship

**DOI:** 10.1136/jme-2024-109912

**Published:** 2024-05-15

**Authors:** Neil Levy

**Affiliations:** 1 Philosophy, Macquarie University, Sydney, New South Wales, Australia; 2 Oxford Uehiro Centre for Practical Ethics, University of Oxford, Oxford, UK

**Keywords:** Ethics, Ethics- Research

## Abstract

The Committee on Publication Ethics (COPE) maintains that AIs (artificial intelligences) cannot be authors of academic papers, because they are unable to take responsibility for them. COPE appears to have the *answerability* sense of responsibility in mind. It is true that AIs cannot be answerable for papers, but responsibility in this sense is not required for authorship in the sciences. I suggest that ethics will be forced to follow suit in dropping responsibility as a criterion for authorship or rethinking its role. I put forward three options for authorship: dropping responsibility as a criterion for authorship, retaining it and excluding AIs, but at the cost of substantial revision of our practices, or requiring only local responsibility for an intellectual contribution.

 AI (artificial intelligence systems, principally large language models like ChatGPT) is rapidly being adopted across all segments of academia (as it is across much of society). The landscape is rapidly changing, and we have yet to settle on the norms that should govern how it is used. Given how extensive usage already is, and how deeply integrated into every aspect of paper production, one important question concerns whether an AI can play the authorship role. Should AIs be credited, in the same way as humans might be, for their contribution to academic research?

While the criteria for authorship are themselves not entirely settled (and differ from discipline to discipline), it is clear that AIs like ChatGPT are already playing roles that were they played by humans would in practice qualify them for authorship at many reputable journals.^1^ In the sciences in particular, knowledge production is distributed across a large number of people, who play different roles.^2^ One person might come up with the initial hypothesis, another might refine that hypothesis, a third might be involved in data analysis or data gathering, and yet others might chip in on the interpretation of the data. AIs are already playing some of these roles.

Just for a single example, AIs are currently asked to generate objections or rebuttals to arguments that the (human) authors intend to make, and even to generate rebuttals to these rebuttals.^3 4^ Sometimes they do this task well. If a human were to do these tasks, they would earn coauthorship in some contexts at least at many reputable journals. So why shouldn’t large language models like ChatGPT or Claude count as authors, too?

The Committee on Publication Ethics (COPE) is perhaps the most influential body attempting to promulgate appropriate ethical standards in academic publication. COPE’s position is that AI may be used in academic research leading to publication, so long as the (human) authors are transparent in their manuscript how the AI was used. But authorship is closed to AIs. ‘AI tools cannot meet the requirements for authorship as they cannot take responsibility for the submitted work.’^5^

What is it to take responsibility for submitted work? Presumably, COPE has in mind the *answerability* sense of responsibility.^6^ To take responsibility for the work in this sense is to be able to answer for it, to be in a position to assert its conclusions and to stand behind them as generated by reliable methods. Those who take responsibility for submitted work are those who deserve credit for its findings and its methods, and perhaps blame if it is shoddy. They are also the people who are legally liable if it contains plagiarism, libel, or in some other way falls short of legal standards.

It’s true that AIs cannot take responsibility for submitted work, in the answerability sense of responsibility. But in contemporary science, it is *normal* for coauthors not to be able to take responsibility for the paper. Typically, there’s a lead author or a small group of leads, and they are able responsibly to assert the conclusions, and they are liable for its failings. But many other authors are not in this position. They may not even have much understanding of the hypothesis being explored: their expertise might be in data analysis, for example.

With regard to a rapidly increasing number of significant publications, *no one*, not even a lead author, is able to take responsibility for the intellectual contributions paper as a whole. The *International Committee of Medical Journal Editors* (ICMJE) sets down demanding criteria for authorship.^7^ Authorship requires (1) substantial contribution to the conception or design of the work, (2) drafting or reviewing the intellectual content of the work, (3) final approval of the version to be published and (4) agreement to be accountable for all aspects of the work, in ensuring that questions related to the integrity and accuracy of the work are appropriately investigated and resolved. An author should also be able to identify which coauthors are responsible for specific other parts of the work, and they must have confidence in the integrity of other authors’ contributions. These criteria are conjunctive: an author must satisfy all of them. It’s clear that right now and in the near future, AIs cannot satisfy these criteria. But it is also clear that a great many human beings who are currently credited with authorship at medical journals (including at least some that are members of the ICMJE) cannot satisfy these criteria either.

A great deal of medical research is ‘radically collaborative’.^8^ It is often the case that *no one* is able to be accountable for radically collaborative research, if accountability requires that the person be able to answer for its integrity and accuracy. No one has an overall grasp of the intellectual contribution the paper makes *and* a grasp of the way in which each component that contributes to that goal functions. No one is able to verify that all the data were collected and analysed appropriately, and that it makes the contribution to the overall picture claimed for it. Some sciences use models that have developed over time in ways that are now beyond retrieval, with the result that no one can responsibly answer for the model, or—correlatively—for the research produced on its basis.^9^ Kukla has pointed out that sometimes ghost writers seem no worse positioned to take responsibility for a paper than the real authors might have been.^10^

Perhaps the ICMJE has sometimes less demanding in mind by its accountability criterion. It requires accountability in ‘ensuring that questions related to the accuracy or integrity of any part of the work are appropriately investigated and resolved’. Perhaps it does not have in mind the capacity to account for accuracy or integrity, but the capacity to establish procedures to check the accuracy or integrity of any part of the work. Understood in that way, the condition is too *un*demanding. There’s no reason why a person with such a capacity should be involved in the research at all: a research coordinator seems better placed to do this sort of work.

It is worth noting that those listed as authors of radically collaborative papers often do not satisfy the other criteria the ICMJE sets down either. Authors who make important intellectual contributions to the work at one site of a trial distributed across multiple geographic areas may not be able to contribute to the overall research project. Their indispensable contribution may depend on expertise no one else in the research group possesses, but that expertise may not allow them to contribute or even usefully comment on other aspects of the project. Indeed, it is in part because expertise is local that those who make important contributions to a paper may be unable to be accountable for it.^8 10^

While research in ethics is rarely as deeply distributed as research in the sciences, the rise of empirical ethics and the increasing use of experimental methods ensures that the same issues may arise in ethics, too. The more contributors are involved in paper production, and the more specialised their roles, the greater the likelihood that some of them will satisfy the criteria for authorship^11^ without being in a position to take responsibility for the paper as a whole.

If the forgoing reflections are accurate, we face a choice between three options. First, we might abandon accountability as a criterion for authorship. We might make our implicit practice explicit and grant authorship to those who make a substantial intellectual contribution to a paper. If we do that, then we may be forced to acknowledge that AIs can be, and already should be, authors.

Second, we might apply the criteria for authorship that the ICMJE sets down, excluding *both* AIs and a great many humans who are currently recognised as authors from that status. We might develop a category of ‘authorship-lite’ to acknowledge those who have made a significant intellectual contribution to a paper but who fall short of being accountable for it. Authorship would be reserved for those who are able to grasp both the overall intellectual contribution of a paper and enough of each of its components to vouch for its accuracy and integrity. In order to qualify as an author, an agent might have to make the sort of contribution to a paper that an author makes to a single-authored paper. Since many people currently regarded as authors do not make this sort of contribution, they could be relegated to a second tier.

While that proposal is not without merit, it has counterintuitive implications in radically collaborative research. It will frequently be the case that *no one* satisfies the authorship role with regard to such research. Radically collaborative research is increasingly common^12^ and represents a large proportion of the most consequential research. Withholding authorship from those who contribute to it might lead to a de-emphasis on authorship altogether. It might come to be seen as a less important status. Perhaps that is as it should be: perhaps it is time to recognise that increasingly authors are not in a position to take responsibility for their papers and that accountability is a matter for the scientific community as a whole, not for individual authors.

Third, we might retain an accountability criterion but apply it locally. If we adopt this option, to qualify for authorship someone would have to make a significant intellectual contribution to a paper *and* be accountable for that contribution (alone). This sort of model is the least revisionary: those people who currently qualify for authorship in practice seem to satisfy these criteria. AIs cannot satisfy these criteria, now or for the foreseeable future, since they are unable to take responsibility for their intellectual contribution to a paper. This option would allow authorship to continue to play its characteristic role in academia (assigning credit in ways that matter for hiring, promotion and grants for example).

Whatever option we take, the confrontation with AIs should force us to reconsider our criteria for authorship. Either authorship cannot require accountability for the paper as a whole, or there are far fewer authors of scientific research than we think. Whatever option we adopt, we must confront the fact that authors often cannot be asked to take responsibility for their papers as a whole.

## Data Availability

There is no data in this work.
